# Introduction to the special collection: The expression of the emotions in man and animals

**DOI:** 10.1017/ehs.2022.59

**Published:** 2022-12-21

**Authors:** Louise Barrett

**Affiliations:** University of Lethbridge, Lethbridge, Alberta, Canada T1K 3M4

*The Expression of the Emotions in Man and Animals* was published on 27 November 1872. It was one of Darwin's speediest publications, written in a four-month period between checking the proofs of *The Descent of Man* and preparing the sixth (and final) edition of *The Origin of Species* (Browne, 2014). As such – as Janet Browne has pointed out – the key to *The Expression of the Emotions* is to recognise that it is very much a sequel to *The Descent* (Browne, 2014). Indeed, it was originally intended to be just a single chapter in the latter, but Darwin had amassed such a large amount of material that a book-length survey seemed preferable – not least because he was aware of how much controversy and criticism *The Descent* was likely to generate. *The Expression of the Emotions* was therefore a way steal a march on his prospective critics, and show that ‘even the most “human” of human characteristics were, at root, derived from animals’ (Browne, 2014: 309).

Not only did *The Expression of the Emotions* further advance Darwin's arguments that humans were an evolved species, but the book also represented an advance in scientific illustration – the book contains a number of photographs that captured the play of expression across a face in a single frozen image. These images allowed Darwin to pinpoint the particular patterns of muscular contraction that were key to our recognition of our emotional expressions, and offered him the means to draw clear parallels across the animal kingdom.

In recognition of *The Expression of the Emotions*’ 150th anniversary, *EHS* decided to put together a special collection of articles to both celebrate Darwin's achievement and to take stock of how the scientific study of emotions has developed since Darwin's day.

To begin our collection, we offer three papers that provide an update on Darwin's original work, and take us in exciting new directions. First, Graziano (2022) gives us a new perspective on the origins of smiling, laughing and crying, based on his own fascinating work on the neurobiology of defensive reflexes and peri-personal space. This is followed by Perrett's (2022) illuminating discussion of the technological advances that have shaped how we represent and study human expressions, and his (often worrying) suggestions for what the future might hold. We then move into unchartered territory for Darwin, with Roberts et al. (2022) offering their insights into a source of emotional expression that does not appear in the 1872 volume: human odour.

From here, we move to a consideration of other animals besides ourselves. Kavanagh et al. (2022) evaluate the evidence for a shared anatomical basis for facial expressions in humans and other primates, while Albuquerque and Resende (2022) tackle the issue of inter-species emotional communication, asking whether domestic dogs can use human emotional expressions in a functional way.

We return again to humans for a series of papers that take on the more complex human emotions, our ‘moral sentiments’, as well as considering the evolved function of other psychological expressions. With respect to the former, Landers and Sznycer (2022) offer original insights into the evolution and function of the shame display, and Akdeniz and van Veelen (2021) present evidence to indicate that an emotional commitment to *not* to being selfish can increase fitness. Our next papers move the conversation along in new directions, with Hunt and Jaeggi (2022) making a case for ‘specialised minds’ that can explain some of the variance we see in personality and psychopathological traits, while Ene et al. (2022) ask whether ‘it is good to be bad?’, and review the evidence for psychopathy as an adaptive trait. Finally, Denault and Zloteneau (2022) critically assess the manner in which Darwin's insights into emotional expression are used (and often misued) by professional ‘body language experts’. They conclude with a plea for evolutionary scientists to both recognise, and help remedy, the spread of misinformation on non-verbal behaviour.

We hope you enjoy reading our special collection and gain a new appreciation of Darwin's original insights and innovative approach. We would like to thank all our authors for their willingness to contribute to our collection and to honour Darwin's legacy so splendidly.
